# Resveratrol Synergistically Promotes BMP9-Induced Osteogenic Differentiation of Mesenchymal Stem Cells

**DOI:** 10.1155/2022/8124085

**Published:** 2022-07-25

**Authors:** Yonghui Wang, Chao Xia, Yang Chen, Tianyuan Jiang, Yan Hu, Yanhong Gao

**Affiliations:** ^1^Department of Geriatrics, Xinhua Hospital, Shanghai Jiaotong University School of Medicine, Shanghai, China; ^2^Department of Geriatrics, Shanghai General Hospital, Shanghai Jiaotong University School of Medicine, Shanghai, China

## Abstract

**Background:**

Mesenchymal stem cells (MSCs) differentiate into osteocytes, adipocytes, and chondrocytes. Resveratrol and bone morphogenetic protein 9 (BMP9) are known osteogenic induction factors of MSCs, but the effect of both resveratrol and BMP9 on osteogenesis is unknown. Herein, we explored whether resveratrol cooperates with BMP9 to improve osteogenic induction.

**Methods:**

The osteogenic induction of resveratrol and BMP9 on C3H10T1/2 cells was evaluated by detecting the staining and activity of the early osteogenic marker alkaline phosphatase (ALP). In addition, the late osteogenic effect was measured by the mRNA and protein levels of osteogenic markers, such as osteopontin (OPN) and osteocalcin (OCN). To assess the bone formation function of resveratrol plus BMP9 *in vivo*, we transplanted BMP9-infected C3H10T1/2 cells into nude mice followed by intragastric injection of resveratrol. Western blot (WB) analysis was utilized to elucidate the mechanism of resveratrol plus BMP9.

**Results:**

Resveratrol not only enhanced osteogenic induction alone but also improved BMP9-induced ALP at 3, 5, and 7 d postinduction. Both the early osteogenic markers (ALP, Runx2, and SP7) and the late osteogenic markers (OPN and OCN) were significantly increased when resveratrol was combined with BMP9. The fetal limb explant culture further verified these results. The *in vivo* bone formation experiment, which involved transplanting BMP9-overexpressing C3H10T1/2 cells into nude mice, also confirmed that resveratrol synergistically enhanced the BMP9-induced bone formation function. Resveratrol phosphorylated adenosine monophosphate- (AMP-) activated protein kinase (AMPK) and stimulated autophagy, but these effects were abolished by inhibiting AMPK and Beclin1 using an inhibitor or siRNA.

**Conclusions:**

Resveratrol combined with BMP9 significantly improves the osteogenic induction of C3H10T1/2 cells by activating AMPK and autophagy.

## 1. Introduction

In adults, bone remodeling is a physical process that balances osteoblast bone formation and osteoclast bone resorption [1]. However, when the balance is disturbed with active bone formation, osteosclerosis occurs, and when the balance is disrupted with active bone resorption, osteoporosis (OP) occurs. OP is characterized by loss of bone mass, deconstruction of microstructure, and susceptivity of fracture [2]. OP is a worldwide health issue affecting hundreds of millions of people, and it creates further health problems, such as pain, disability, infection, and even death. In OP, adipogenesis in the bone marrow is increased at the expense of bone formation, leading to bone loss and fat accumulation in the bone marrow. Mesenchymal stem cells (MSCs) were first discovered in the bone marrow, and they can undergo osteogenic or adipose lineage commitment. MSCs commit to differentiate into osteoprogenitor cells and then into preosteoblasts, which eventually become mature osteoblasts [3]. Many physical, chemical, and biological factors take part in MSC differentiation. In recent decades, numerous studies have uncovered a number of essential pathways that are involved in regulating the lineage commitment of MSCs, including transforming growth factor-beta (TGF-*β*)/bone morphogenetic protein (BMP) signaling, wingless-type MMTV integration site (Wnt) signaling, Hedgehog (Hh) signaling, Notch signaling, and fibroblast growth factor (FGF) signaling [3]. Thus, the study of osteoblast lineage commitment of MSCs may provide important information for OP treatment in the future.

BMPs belong to the TGF-*β* family, and they are widely involved in regulating cell proliferation, cell differentiation, and embryonic development [4]. Interestingly, BMP signaling is also involved in embryonic skeletogenesis and maintenance of postnatal bone. Among the 14 BMPs, BMP2, BMP4, BMP5, BMP7, and BMP9 are widely studied and have been demonstrated to exhibit high osteogenic activity [5–9]. Many clinical trials have indicated that BMP2 and BMP7 have promising therapeutic potential in fracture healing and spine fusion, suggesting that BMPs have potential therapeutic applications for bone disorders [10, 11]. Moreover, BMP9 (also called growth differentiation factor 2 (GDF2)) is involved in liver fibrosis, heart failure, tumors, glucose metabolism, and lipid metabolism [12–14]. In osteodifferentiation of MSCs, BMP9 has been demonstrated to induce osteoblast differentiation and suppress osteoclast differentiation both *in vitro* and *in vivo* via a phosphorylating the Smad-dependent pathway or via the Smad-independent pathway [15]. As an effective factor to induce osteogenic differentiation, a large cross-talk network has been discovered between BMP9 and the Wnt/*β*-catenin, Hh, FGF, TGF-*β*, and Notch signaling pathways [15–19]. Moreover, to further enhance the osteogenic induction of BMP9, researchers focused on combination therapy that associates BMP9 and other osteogenic inducers. For instance, follicle-stimulating hormone *β*-subunit, retinoic acid, FK506, and emodin were proved to play a synergistic role in BMP9-induced osteogenesis [20–23]. In our previous research, also, we found that melatonin and triiodothyronine potentiate BMP9-induced osteogenic differentiation of MSCs [24, 25]. Furthermore, the traditional regiments of OP, including bisphosphonates, calcitonin, selective estrogen response modulators, and estrogen, reduce fractures significantly but have rare and severe adverse effects [26]. Due to the multiple differentiation and self-renew ability, MSC transplantation has been considered to be applied in osteoporosis. The abovementioned researches uncovered the clue that the combined strategy, consisting BMP9 and other osteogenic inducers, would induce stronger osteogenic differentiation of MSCs, which would be a promising treatment of osteoporosis in the future.

Resveratrol, also known as 3,5,4′-trihydroxy-trans-stilbene, is a polyphenolic phytoestrogen and phytoalexin product in plants, such as grapes, berries, and peanuts. Resveratrol has a variety of potentially beneficial health effects and has been reported to possess anti-inflammatory, immunomodulatory, and antioxidative capacities [27, 28]. Resveratrol has been shown to stimulate the proliferation and differentiation of MC3T3 osteoblasts and inhibit osteoclast differentiation [29, 30]. Similarly, *in vivo* studies have demonstrated that resveratrol increases bone mineral density (BMD) in ovariectomized (OVX) female rat models [31, 32]. It is widely acknowledged that the osteogenic effect of resveratrol depends on its estrogen-like effect and activation of sirtuin-1 (SIRT1) [33]. Further studies have indicated that resveratrol protects osteoblasts against dexamethasone-induced osteogenesis suppression by activation of the adenosine monophosphate- (AMP-) activated protein kinase (AMPK) pathway, and it promotes osteoblastic differentiation in the OVX OP model by regulating autophagy [34]. However, the interactions between resveratrol and BMP9 remain unknown.

The present study is aimed at investigating the effect of resveratrol on BMP-induced osteogenesis in C3H10T1/2 cells and the potential molecular mechanism. Our results revealed that resveratrol promotes BMP9-induced osteogenic differentiation through activation of the AMPK/Autophagy signaling pathway. Thus, resveratrol is an effective enhancer of BMP9, suggesting that it may lead to novel therapies for OP.

## 2. Methods

### 2.1. Cell Culture and Reagents

C3H10T1/2 cells were purchased from Shanghai Institutes for Biological Science (Shanghai, China). Cells were cultured in Dulbecco's modified Eagle's medium (DMEM) (Hyclone, USA), containing 10% fetal bovine serum (FBS) (Gibco, USA), 100 U/mL penicillin, and 100 mg/mL streptomycin (Gibco, USA), at 37°C in 5% CO_2_. Resveratrol (Sigma, USA) was diluted to 50 mM for storage in dimethylsulfoxide (DMSO) (Sigma, USA). Osteogenic differentiation was induced when cells reached 70% confluence, and the osteogenic induction medium, consisting of DMEM, 10% FBS, 10 mM *β*-glycerophosphate, 50 *μ*g/L L-ascorbic acid, and 100 nM dexamethasone, was replaced every 3 d (Sigma, USA). Ad-BMP9 and Ad-Green fluorescent protein (GFP) were obtained from HanBio. Before infection, cells were plated in 6-well plates. And when the cells reached 80% confluence, add Ad-BMP9 or Ad-GFP and polybrene (Yeason, China) 8*μ*g/*μ*L for 6 h. The medium was then changed to complete medium.

### 2.2. Alkaline Phosphatase (ALP) Activity and Staining

Cells were infected with Ad-GFP or Ad-BMP9 with or without resveratrol for 3, 5, and 7 d. At the indicated time points, cells were lysed with RIPA lysis buffer (Beyotime, China) and centrifuged at 12000 × g for 10 min. The supernatant was collected to measure the ALP activity and protein concentration with Alkaline Phosphatase Assay and BCA Kit, respectively (Beyotime, China). The final ALP activity was normalized to total intercellular protein content according to the manufacturer's instructions.

For ALP staining, C3H10T1/2 cells were seeded into 12-well plates, and cells were transduced with Ad-GFP or Ad-BMP9 in the presence or absence of resveratrol. After 7 d of osteogenic differentiation, the medium was removed, and cells were washed three times with PBS. Cells were fixed with 4% paraformaldehyde (Beyotime, China) for 15 min at room temperature and then stained using the BCIP/NBT Alkaline Phosphatase Staining Assay Kit (Beyotime, China) according to the manufacturer's protocol. Images were captured on a Leica 300 DMI microscope.

### 2.3. Alizarin Red S Staining

Alizarin red S staining was utilized to evaluate the level of matrix mineralization. Osteogenic differentiation was induced in C3H10T1/2 cells as previously described. After 14 d of induction, cells were fixed with 4% paraformaldehyde for 15 min at room temperature, washed three times with PBS, and then stained with 2% Alizarin red S (Sigma, USA) for 30 min at room temperature. Images were captured on a Leica 300 DMI microscope.

### 2.4. Immunohistochemical Staining

After 21 d of osteogenic differentiation, cells were fixed with 4% paraformaldehyde for 15 min at room temperature and washed with PBS. Cells were then permeabilized with 0.1% Triton-X (Sigma, USA) for 15 min and blocked with 5% BSA for 1 h (Beyotime, China) at room temperature. Cells were incubated overnight at 4°C with the following primary antibodies: OCN (sc30045, Santa Cruz Biotechnology) and OPN (ab91655, Abcam). Cells were then washed with PBS and incubated with a biotin-labeled secondary antibody (ABclonal, China) for 1 h at room temperature. Cells were then stained with diaminobenzidine (DAB) (Beyotime, China) to detect the protein and then visualized using a microscope (Olympus BX51, Japan).

### 2.5. Quantitative Real-Time PCR (qRT-PCR)

Total RNA from cells induced for 7 d was extracted with TRIzol (Takara, Japan) as previously described [35]. cDNA was synthesized using PrimeScript RT Master Mix (TAKARA, Japan), and qRT-PCR was performed using SYBR Green Master Mix (Yeason, China). The relative expression level of each target gene was calculated using the 2^−*ΔΔ*Ct^ method with GAPDH as a loading control.

The following primers were used: ALP forward, 5′-GACTGGTACTCGGATAACGA-3′; ALP reverse, 5′-TGCGGTTCCAGACATAGTGG-3′; SP7 forward, 5′-CAAAGAAGCCATACGCTGAC-3′; SP7 reverse, 5′-GTCCATTGGTGCTTGAGAAG-3′; Runx2 forward, 5′-TGAGGGATGAAATGCTTGGGAACTG-3′; Runx2 reverse, 5′-GATGATGACACTGCCACCTCTGAC-3′; GAPDH forward, 5′-AGGTCGGTGTGAACGGATTTG-3′; GAPDH reverse, 5′-TGTAGACCATGTAGTTGAGGTCA-3′.

### 2.6. Western Blot (WB) Analysis

Cells were seeded in 6-well plates and treated according to the experimental design. Total protein was obtained after cell lysis with RIPA lysis buffer (Beyotime, China), and cleared lysates were denatured by boiling for 10 min. Proteins were then separated with 10% sodium dodecyl sulfate-polyacrylamide gel electrophoresis (SDS-PAGE) (ABclonal, China) and then transferred to polyvinylidene difluoride (PVDF) membranes (Millipore, USA). The membranes were blocked with fast block buffer (Epizyme, China) for 20 min at room temperature. The membranes were then incubated overnight at 4°C with the following primary antibodies: AMPK (2532, Cell Signaling Technology), p-AMPK (2535, Cell Signaling Technology), Beclin1 (3495, Cell Signaling Technology), p62 (sc-28359, Santa Cruz Biotechnology), LC3 (14600-1-AP, Proteintech), and alpha-Tubulin (66031-1-Ig, Proteintech). After washing, the membranes were then incubated with horseradish peroxidase- (HRP-) conjugated goat anti-mouse and goat anti-rabbit antibodies (AS014 and AS003, ABclonal) for 1 h at room temperature.

### 2.7. Transient Transfection with Small Interfering RNAs (siRNAs)

C3H10T1/2 cells were plated in 6-well plates and then transfected with AMPK*α*1/2 siRNA or control siRNA (GenePharma, China) using Lipofectamine 2000 transfection reagent (Invitrogen, USA) according to the manufacturer's instructions.

The siRNA sequences were as follows: AMPK*α* siRNA, 5′-AAGAGAAGCAGAAGCACGACG-3′; control, 5′-AAGCCGGTATGCCGGTTAAGT-3′.

The siRNAs for Beclin1 were designed by RiboBio (Guangzhou). The siRNA sequence of Beclin1 is as follows: 5′-CCTGTGGAGTGGAATGAAA-3′.

### 2.8. Fetal Limb Explant Culture

Fetal limb explant culture was performed based on previously described procedure [24, 25]. Fetal limbs were dissected from mouse embryos (E18.5) under sterile conditions and cultured in DMEM containing 0.5% BSA, 50 *μ*g/mL ascorbic acid, 1 mM *β*-glycerophosphate, and 100 mg/mL penicillin and streptomycin, at 37°C in 5% CO_2_. After 24 h of *in vitro* culture, the limbs were treated accordingly. The medium was changed every 3 d, and calcein (100 mM, Sigma, USA) was added to the medium 2 d before the endpoint. After 12 d of culture, the skin and muscle on the fetal limbs were removed, and new bone formation was assessed using fluorescence microscopy and histology. At least five limb explants were included in each group. Images were captured on a Leica 300 DMI microscope.

### 2.9. C3H10T1/2 Implantation and Microcomputed Tomography (*μ*CT) Analysis

The subcutaneous implantation of C3H10T1/2 cells and the induction of ectopic bone formation were performed as previously described [36]. Cells were infected with Ad-GFP or Ad-BMP9 for 7 d and then subcutaneously injected (5∗10^6^ cells per injection) into male athymic nude mice (5 per group; 4–6 weeks old; Shanghai Laboratory Animal Centre, China). Resveratrol (20 mg·g-1/d) or PBS was intragastrically administered to the mice for 5 weeks. The mice were then euthanized, and the implanted cells were harvested for *μ*CT scans (VENUS 001; PINGSENG Healthcare, China) at a voltage of 90 kV, beam current of 0.07 mA, and resolution of 20.0 *μ*m. The images were analyzed using Avatar3 software (PINGSENG Healthcare). Two-/three-dimensional images were obtained and analyzed to evaluate bone mass, bone mineral density (BMD), and bone volume/total volume (BV/TV) of the masses. All experiments were performed in accordance with the guidelines for animal experimentation of the Ethics Committee of Xinhua Hospital.

### 2.10. Histological Staining

After fixing with 4% paraformaldehyde overnight, the samples were decalcified and dehydrated for 4 weeks. The samples were then embedded in paraffin (Beyotime, China); sections (4 *μ*M) were obtained from paraffin blocks using a microtome (Leica Biosystems, Wetzlar, Germany) and stained with hematoxylin and eosin (H&E) and Masson's trichrome staining as previously described [37]. The histological sections were observed under an optical microscope (Olympus BX51, Japan).

### 2.11. Statistical Analysis

All results are expressed as the mean ± standard deviation (SD). Each experiment was repeated at least three times. Student's *t*-test was used for the variable comparisons between two groups by GraphPad Prism 8.3.0 software (GraphPad, USA). *P* values < 0.05 were considered statistically significant.

## 3. Results

### 3.1. Resveratrol Synergistically Promotes BMP9-Induced Osteogenic Differentiation of MSCs

**Figure 1:**
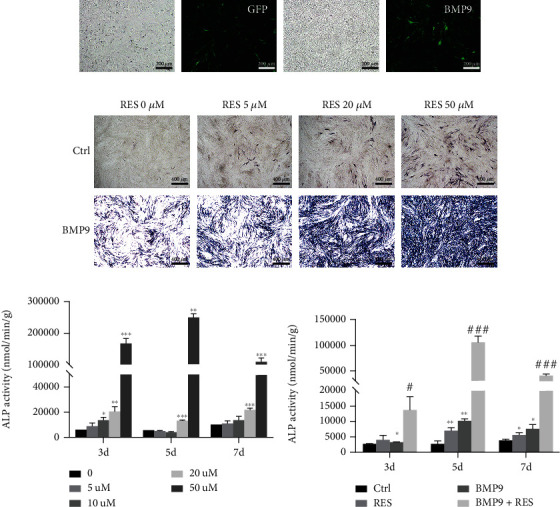
Resveratrol enhances the BMP9-induced early osteogenic marker, ALP, in C3H10T1/2 cells. (a) C3H10T1/2 cells were infected with Ad-GFP and Ad-BMP9, and the fluorescence was observed under a microscope to determine the adenoviral infection efficiency after 48 h of infection. (b) The osteogenic induction of 5 *μ*M, 20 *μ*M, and 50 *μ*M resveratrol with Ad-GFP or Ad-BMP9 was determined by ALP staining after 7 d of osteogenic differentiation. (c) The osteogenic differentiation of C3H10T1/2 cells with different concentrations of resveratrol (0, 5, 10, 20, and 50 *μ*M) for 3, 5, and 7 d was quantitatively measured with ALP activity. (d) Resveratrol (50 *μ*M) potentiated BMP9-induced osteogenic differentiation, and the early osteogenic marker, ALP, was measured by ALP activity. ^∗^*P* < 0.05, ^∗∗^*P* < 0.01, and ^∗∗∗^*P* < 0.001 compared to the control group. ^#^*P* < 0.05, ^##^*P* < 0.01, and ^###^*P* < 0.001 compared with the BMP9 group.

First, we verified the infection efficiency of Ad-BMP9 (BMP9) and Ad-GFP (Ctrl) adenoviral vectors in C3H10T1/2 cells (Figures 1(a) and S1A). To explore the osteogenic effect of resveratrol on C3H10T1/2 cells, ALP staining and ALP activity assays were performed. As shown in Figures 1(b) and 1(c), resveratrol promoted osteoblast differentiation at concentrations ranging from 5 *μ*M to 50 *μ*M at days 3, 5, and 7 postinduction. At 100 *μ*M, however, resveratrol induced osteoblast cell death (data not shown). Thus, we selected a resveratrol concentration of 50 *μ*M for the subsequent experiments. Resveratrol alone promoted osteogenesis, but its osteogenic effect was enhanced in the BMP9-infected group (Figure 1(b)). Consistently, the ALP activity was higher in the BMP9+resveratrol (BMP9+RES) group compared to the BMP9-infected group (Figure 1(d)). Thus, these findings indicated that resveratrol combined with BMP9 significantly enhances ALP activity in C3H10T1/2 cells.

### 3.2. Resveratrol Enhances BMP9-Induced Osteogenic Marker Expression Levels and Matrix Mineralization

**Figure 2:**
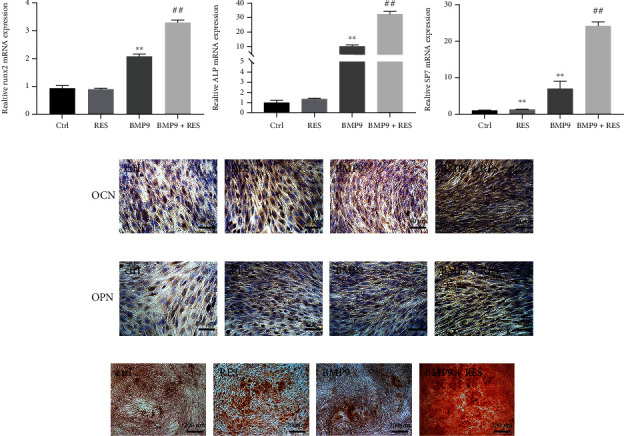
Resveratrol promotes BMP9-induced osteogenic marker expression and matrix mineralization in C3H10T1/2 cells. (a) The mRNA expression levels of early osteogenic markers (ALP, Runx2, and SP7 mRNA) were highest in the BMP9+resveratrol group after 7 d of induction. (b, c) Immunohistochemical staining of late osteogenic markers (OCN and OPN) in C3H10T1/2 cells after 14 d of induction. (d) Alizarin red S staining of C3H10T1/2 cells after 21 d of osteogenic differentiation. ^∗^*P* < 0.05, ^∗∗^*P* < 0.01, and ^∗∗∗^*P* < 0.001 compared to the control group. ^#^*P* < 0.05, ^##^*P* < 0.01, and ^###^*P* < 0.001 compared with the BMP9 group.

To further investigate the synergistic ability of resveratrol and BMP9 to induce osteogenesis, early and late osteogenic differentiation indexes were analyzed. The mRNA expression levels of ALP, Runx2, and SP7 were significantly increased in the BMP9+resveratrol group compared to the control, resveratrol, and BMP9 groups 7 d after osteogenic induction (Figure 2(a)). Moreover, after 14 d of osteogenic differentiation, the protein levels of osteopontin (OPN) and osteocalcin (OCN) were higher in the BMP9+resveratrol group compared to the other three groups as shown by immunohistochemical staining (Figures 2(b) and 2(c)). Additionally, Alizarin red S staining showed that the mineral nodule formation was enhanced in the BMP9+resveratrol group compared to the other groups when C3H10T1/2 cells were differentiated for 21 d (Figure 2(d)). In summary, these results demonstrated that resveratrol combined with BMP9 significantly improves osteogenesis in MSCs.

### 3.3. The Combination of Resveratrol and BMP9 Promotes Bone Formation in Mouse Embryo Limb Explant Culture

**Figure 3:**
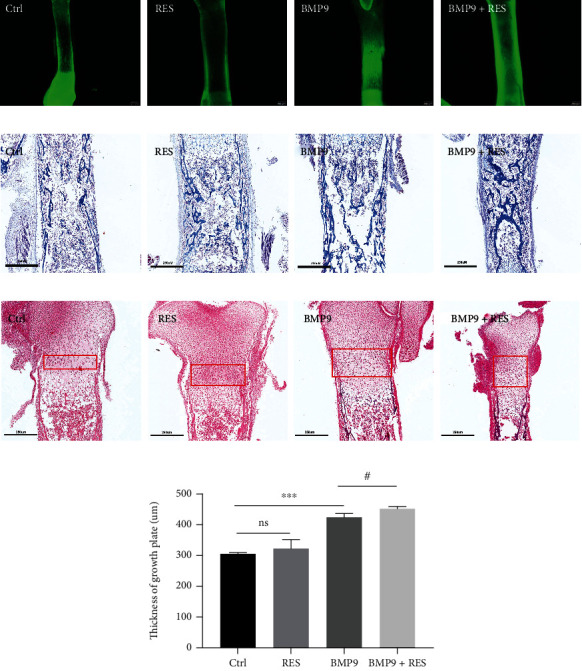
Resveratrol promotes the expansion of the BMP9-induced hypertrophic chondrocyte zone. (a) Micrographic fluorescent images of fetal mouse tibia from E18.5 embryos treated with Ad-GFP, Ad-GFP+resveratrol, Ad-BMP9, or Ad-BMP9+resveratrol for 14 d. *n* = 5. (b, c) The *in vitro* osteogenic induced fetal mouse tibias were fixed and stained with H&E and Masson's trichrome staining. The boxes indicate the growth plate. (d) Analysis of the thickness of growth plates. The slides were scanned and measured the distance from the proliferation zone to the hypertrophic zone which is the growth plate, by K-Viewer software (KFBIO, China). ^∗^*P* < 0.05, ^∗∗^*P* < 0.01, and ^∗∗∗^*P* < 0.001 compared to the control group. ^#^*P* < 0.05, ^##^*P* < 0.01, and ^###^*P* < 0.001 compared with the BMP9 group.

We next aimed to analyze the effect of resveratrol on developing bone by using a fetal limb culture assay. The limbs of E18.5 mouse embryos were isolated and divided into the following four groups: GFP (Ctrl), GFP+resveratrol (RES), BMP9 (BMP9), and BMP9+resveratrol (BMP9+RES). After culturing for 14 d, a calcein fluorescent dye was utilized to indicate new bone formation with different fluorescence intensities. As shown in Figure 3(a), the BMP9+resveratrol treatment significantly promoted new bone formation as indicated by the strongest fluorescence intensity compared to the other three groups. The epiphyseal growth plate is the main site of longitudinal growth of the long bones. The cartilage at this site is formed by the proliferation and hypertrophy of cells and synthesis of typical extracellular matrix. The formed cartilage is then calcified, degraded, and replaced by osseous tissue. Thus, chondrogenesis of growth plates will influence the endochondral bone formation [21, 38]. The histological evaluation using H&E and Masson's trichrome staining showed that the BMP9+resveratrol group had a larger area of trabecular matrix and thicker growth plates, which are indicators of bone formation (Figures 3(b)–3(d)). These results suggested that resveratrol and BMP9 might act synergistically to accelerate chondrocyte hypertrophy and subsequently endochondral bone formation.

### 3.4. Resveratrol Improves BMP9-Induced Ectopic Bone Formation

**Figure 4:**
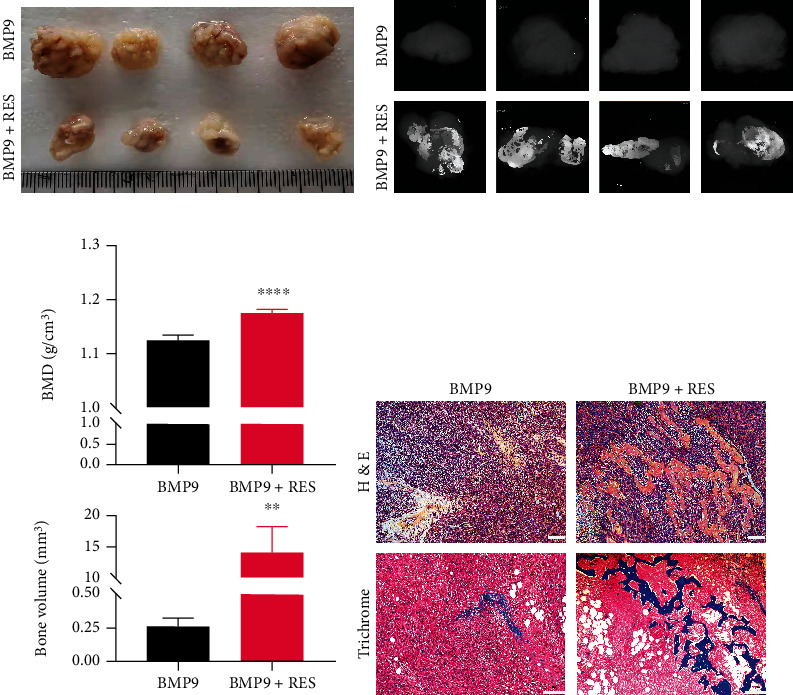
Resveratrol synergistically enhances BMP-induced ectopic bone formation. (a) Macrographic images of BMP9- and BMP9+resveratrol-induced ectopic bones after 5 weeks. (b) *μ*CT images of BMP9- and BMP9+resveratrol-induced ectopic bones. *n* = 4. (c) Quantitative analysis of the BMD and bone volume of ectopic bone by *μ*CT. (d) H&E and Masson's trichrome staining of paraffin-fixed ectopic bones. ^∗^*P* < 0.05, ^∗∗^*P* < 0.01, and ^∗∗∗^*P* < 0.001 compared to the BMP9 group.

To further confirm the facilitating effect of resveratrol on BMP9-induced bone formation, we performed ectopic MSC transplantation *in vivo*. We infected C3H10T1/2 cells with either Ad-GFP or Ad-BMP9 in the presence or absence of 50 *μ*L of resveratrol for 3 d and then subcutaneously injected these cells into BALB/c nude mice, and mice were then subjected to intragastric administration of resveratrol or PBS once every 2 d. After 5 weeks of treatment, mice were sacrificed, and ectopic bones were collected. As shown in Figure 4(a), only the BMP9 and BMP9+resveratrol groups formed larger ectopic masses. However, when analyzing the mineral density and bone volume, the BMP9+resveratrol group had higher BMD and bone volume compared to the other groups (Figures 4(b) and 4(c)). Consistent with *μ*CT analysis, the BMP9+resveratrol group had more bone mineral matrix compared to the BMP9 group as shown by H&E and Masson's trichrome staining (Figure 4(d)). Taken together, these findings indicated that resveratrol facilitates BMP-induced bone mineral formation.

### 3.5. Resveratrol Synergistically Acts with BMP9 to Promote Osteogenic Differentiation via an AMPK-Dependent Pathway

**Figure 5:**
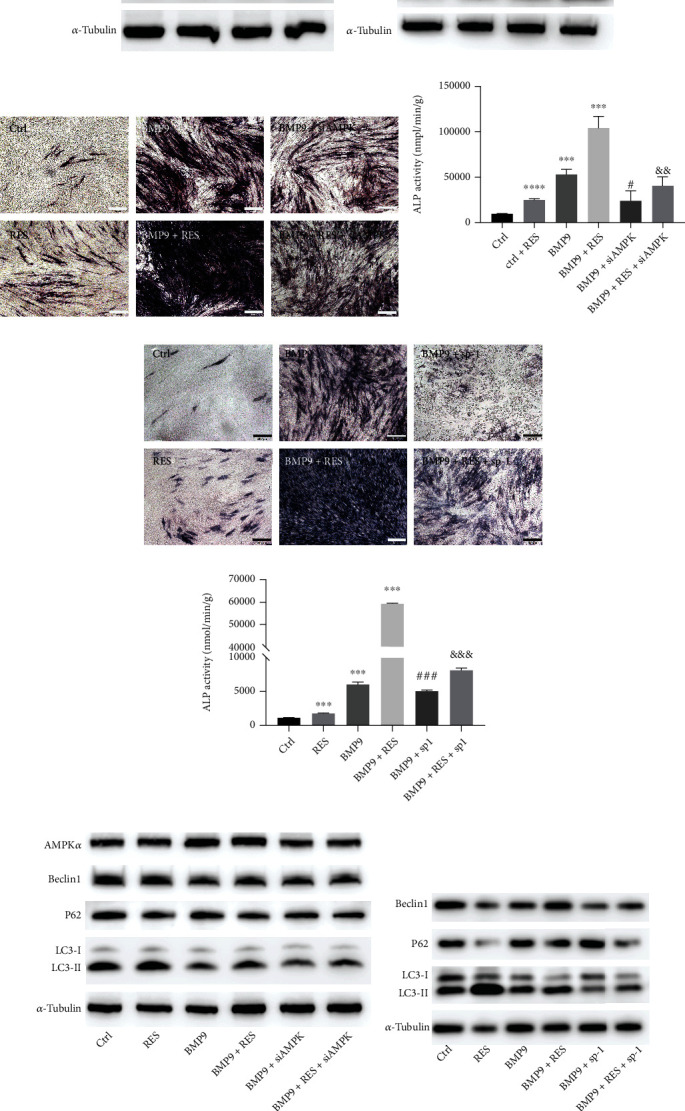
Resveratrol promotes BMP9-induced bone formation by activating the AMPK/autophagy signaling pathway. (a) Cell lysates were collected after infection with Ad-GFP or Ad-BMP9 with or without resveratrol (50 *μ*M) for 48 h. The protein levels of p-AMPK and t-AMPK were measured by WB analysis. (b) Autophagy was induced by adding 50 *μ*M resveratrol to Ad-GFP or Ad-BMP9. The LC3, Beclin1, and p62 autophagy markers were assessed by WB analysis. (d, e) siAMPK*α* inhibited the ALP of BMP9 and BMP9+resveratrol-induced osteogenic differentiation of C3H10T1/2 cells. After pretreatment of C3H10T1/2, cells with siAMPK*α* followed by osteogenic differentiation with BMP9 or BMP9+resveratrol for 7 d, ALP staining (c) and ALP activity assays (d) were performed to measure the osteogenic ability. (e, f) The SP-1 autophagy inhibitor suppressed the osteogenic differentiation in the BMP9 and BMP9+resveratrol groups. Cells were treated with the combination of SP-1 and Ad-BMP9 or Ad-BMP9+resveratrol for 7 d, and ALP staining (e) and ALP activity assays (f) were performed to measure the osteogenic induction in C3H10T1/2 cells. (g) WB analysis showed that pretreatment with siAMPK*α* decreased the AMPK level. C3H10T1/2 cells were transfected with siAMPK*α* 24 h prior to treatment with Ad-BMP9 or Ad-BMP9+resveratrol. After another 48 h, protein lysates were collected to measure AMPK levels by WB analysis. (h) WB analysis showed that autophagy was inhibited by SP-1. After treatment of C3H10T1/2 cells with SP-1 combined with Ad-BMP9 or Ad-BMP9+resveratrol for 48 h, cell lysates were collected to measure the protein levels of LC3, Beclin1, and p62 by WB analysis. ^∗^*P* < 0.05, ^∗∗^*P* < 0.01, and ^∗∗∗^*P* < 0.001 compared to the control group. ^#^*P* < 0.05, ^##^*P* < 0.01, and ^###^*P* < 0.001 compared with the BMP9 group. ^&^*P* < 0.05, ^&&^*P* < 0.01, and ^&&&^*P* < 0.001 compared to the BMP9+resveratrol group.

Resveratrol protects bone against dexamethasone, lipopolysaccharides, and cadmium-induced bone loss. The osteogenic ability of resveratrol depends on activating the AMPK pathway. It is well acknowledged that AMPK induction is critical for bone formation. Thus, we hypothesized that resveratrol promotes bone formation via the AMPK or ERK pathway. Compared with Ctrl or BMP9 alone, AMPK phosphorylation was higher when resveratrol was added to the Ctrl or BMP9 groups, and the total AMPK levels remained the same among the groups (Figure 5(a)). Moreover, we performed ALP staining and ALP activity assays to evaluate whether the osteogenic effect of resveratrol depends on AMPK by utilizing siAMPK*α*. As shown in Figure S2A, siAMPK inhibited the protein levels of total AMPK. After knocking down AMPK expression, the ALP staining and activity levels were decreased compared with those in the BMP9 and BMP9+resveratrol groups (Figures 5(c), 5(d), and 5(g)). As autophagy is regulated by AMPK and resveratrol has been reported to stimulate autophagy, we speculated that resveratrol mediates osteogenic function through autophagy. We found that resveratrol significantly increased the protein levels of LC-II and Beclin1 but decreased the protein levels of p62 (Figure 5(b)). Additionally, mTOR signaling, upstream of autophagy, was inhibited by exposure to resveratrol (Figure S1C). ALP staining and activity were decreased when the BMP9 and BMP9+resveratrol groups were treated with spautin-1 (SP-1), an autophagy inhibitor (Figures 5(e) and 5(f)). Consistently, treatment with SP-1 effectively inhibited autophagy by decreasing the levels of LC-II and Beclin1 compared with the BMP9 and BMP9+resveratrol groups (Figure 5(h)). Additionally, the AMPK inhibitor, compound C, and siBeclin1 suppressed the phosphorylation of AMPK (Figure S2A) and Beclin1 expression (Figure S2B), which further inhibited the ALP staining (Figures S3A and S4A) and activity (Figures S3B and S4B) in the BMP9 and BMP9+resveratrol groups, respectively. Moreover, the phosphorylation and autophagy induced by resveratrol were inhibited by compound C in the BMP9 and BMP9+resveratrol groups (Figure S3C), and autophagy was suppressed by siBeclin1 transfection in BMP9 and BMP9+resveratrol groups (Figure S4C). Together, these results indicated that the resveratrol-mediated osteogenic effect occurs through the AMPK/autophagy pathway.

## 4. Discussion

In the present study, we demonstrated that resveratrol acts synergistically with BMP9 to induce osteogenesis in the C3H10T1/2 MSC line. As known osteogenic inducers, the combination of BMP9 and resveratrol increased the expression of osteogenesis markers. Specifically, at 50 *μ*M, the osteogenesis effect of resveratrol was strongest in C3H10T1/2 cells. Furthermore, when C3H10T1/2 cells were cotreated with BMP9 and resveratrol, the induction of osteogenesis was stronger than resveratrol or BMP9 alone, resulting in higher ALP activity as well as higher mRNA and protein expression levels of bone formation-related markers. The *in vivo* mouse embryo limb explant culture and ectopic MSC transplantation study also verified that the strongest osteogenesis effect resulted from the combination of BMP9 and resveratrol. Moreover, resveratrol phosphorylated AMPK and stimulated autophagy with or without BMP9, suggesting that resveratrol improves BMP9-induced osteogenic differentiation through the AMPK/autophagy signaling pathway. Moreover, inhibition of AMPK and autophagy by treatment with siRNA or inhibitor significantly attenuated ALP activity, further demonstrating that resveratrol is involved in BMP9-induced osteogenesis through the AMPK signaling pathway in MSCs. In summary, our results showed that resveratrol has a distinct osteogenic effect by activating the AMPK/autophagy signaling pathway.

BMP9 is a well-studied BMP, and it has a potent osteogenic induction effect. Overexpression of BMP9 in C3H10T1/2, C2C12, TE85, and MC3T3 cells leads to higher mRNA and protein levels of osteogenesis markers and ALP activity as early as 5 d after overexpression [39]. Compared with other BMPs, such as BMP2 and BMP7, treatment with BMP9 has greater osteogenic induction capacity as indicated by higher levels of osteogenic markers [40, 41]. In addition to viral vectors, overexpression of BMP9 using nonadenoviral vectors, including recombinant BMP9 (rbBMP9) and a peptide derived from BMP9 (pBMP9), increases ALP activity and osteogenic marker expression [42]. *In vivo* research has further demonstrated the osteogenic induction ability of BMP9 via intramuscular injection of BMP9-transduced C2C12 cells in athymic nude mice [43]. The abovementioned studies demonstrate the osteogenic potential of BMP9. Additionally, further research has indicated that various compounds may potentiate the osteogenic induction effect of BMP9. For example, all-trans-retinoid acid induces osteogenic differentiation and cooperates with BMP2 to induce osteogenic differentiation in preadipocytes, and its cooperation with BMP9 shows the same results by activating the BMP/Smad and Wnt/*β*-catenin pathways [44]. Furthermore, the follicle-stimulating hormone *β*-subunit also potentiates BMP9-induced osteogenic differentiation in mouse embryonic fibroblasts [20]. In our previous research, we found that treatment of MSCs with melatonin or triiodothyronine significantly improves BMP9-induced osteogenic differentiation [24, 25]. Thus, the combination of osteogenic factors with BMP9 may be a promising strategy for treating OP in the future.

In 2014, researchers revealed the bone protective function of resveratrol in middle-aged obese men with metabolic syndrome [45]; they showed that oral administration of 1 g/d resveratrol to obese men over 4 weeks results in increased serum bone ALP and higher lumbar spine BMD. Another study, enrolling type 2 diabetes patients, has further confirmed the beneficial effects of high-dose resveratrol on bone health, showing higher calcium concentrations and 25-hydroxy vitamin D as well as prevention of BMD and bone mineral content (BMC) reduction compared to the placebo group [46]. These results are supported by *in vivo* studies demonstrating that resveratrol protects from bone loss. In OVX rats, 25 mg·kg^−1^/d and 45 mg·kg^−1^/d daily doses of resveratrol significantly elevate decreased BMD, and another *in vivo* study has revealed that resveratrol promotes osteoblast differentiation of BMSCs via the SIRT1/NF-*Κ*B signaling pathway [47, 48]. Another study has verified the bone protective function of resveratrol by inhibiting osteoclastogenesis through its antioxidant capability [49, 50] via the PI3K/AKT/FoxO1 signaling pathway. Being an effective treatment for OP in animal OP models, Li et al. coupled resveratrol through a hydrolysable covalent bond with the carboxylic acid groups in porous poly-*ε*-caprolactone (PCL) surface grafted with acrylic acid [51]; these authors used both *in vivo* and *in vitro* experiments to demonstrate that this scaffold significantly facilitates osteogenesis. Despite directly protecting bone loss by increasing osteogenesis and inhibiting osteoclastogenesis, resveratrol has also been demonstrated to inhibit adipogenesis of human bone marrow stromal stem cells to indirectly maintain bone mass [52]. There were researches testifying whether resveratrol is influential to BMPs induced bone formation or not. For instance, Kuroyanagi et al. reported that resveratrol enhances the BMP4-stimulated synthesis of osteoprotegerin (OPG) in osteoblasts via the p38/MAPK signaling pathway [53]. However, a study reported that resveratrol does not enhance the effects of BMP4 on other osteogenic markers, ALP activity, and mineral nodule deposition. In contrast, a previous study has reported that resveratrol does not influence BMP6-induced ALP activity and OCN expression [54], but this study was performed in 2003, suggesting that the results may have been restricted due to technical limitations. According to our results, resveratrol combined with BMP9 resulted in higher expression of osteogenic markers, ALP activity, and mineral nodule deposition than BMP9 alone. In summary, resveratrol synergized with BMP9 to promote osteogenesis of MSCs, which would prompt stem cell transplantation therapy for OP.

AMPK is a heterotrimeric complex that is comprised of a catabolic *α* subunit and regulatory *β* and *γ* subunits. AMPK is widely expressed in various organs, including bone, and it plays a critical role in osteoblast proliferation and differentiation [55]. Resveratrol has been reported to activate the AMPK signaling pathway through SIRT1-dependent and SIRT1-independent pathways [56, 57]. Although resveratrol activated the AMPK signaling pathway in the present study, it failed to activate SIRT1 (Figure S1B). When AMPK was inhibited by compound C or transcriptionally downregulated by siAMPK*α*, the ALP staining and activity were decreased compared to the BMP and BMP9+resveratrol groups. These results indicated that resveratrol promotes BMP9-induced osteogenesis by a SIRT1-independent AMPK signaling pathway. AMPK is involved in various physiological processes, including autophagy, which is a well-acknowledged AMPK-activated process and is essential for the osteogenic differentiation of MSCs and osteoblasts [58, 59]. AMPK activates autophagy by phosphorylating ULK1, which is critical for autophagy initiation [60]. In addition, autophagy can also be induced by phosphorylating the phosphatidylinositol 3-kinase catalytic subunit type 3 complex and Beclin1, which are essential for autophagosome formation [61]. According to Li et al., AMPK stimulates osteoblast differentiation and mineralization by autophagy [58]. Li et al. also showed that compound C, an inhibitor of AMPK, inhibits autophagy and osteogenic differentiation in MC3T3 cells, and they demonstrated that treatment with an autophagy inhibitor or Beclin1 silencing inhibits both autophagy and osteogenic differentiation. Thus, AMPK activation may stimulate osteoblast differentiation and mineralization by inducing autophagy. Additionally, resveratrol has been demonstrated to promote osteogenic differentiation of human gingival mesenchymal stem cells (HGMSCs) by activating AMPK and autophagy [56]; treatment of HGMSCs with 1 *μ*M resveratrol results in AMPK phosphorylation and autophagosome formation, but treatment with SP-1, an autophagy inhibitor, suppresses the resveratrol-induced osteogenic differentiation and autophagy of HGMSCs. Consistent with the abovementioned research, the present study demonstrated that resveratrol induced autophagy in both the control and BMP9-treated groups. Similarly, when the autophagy inhibitor, SP-1, or Beclin1 silencing was applied to the BMP9 and BMP9+resveratrol groups, the osteogenic differentiation was significantly inhibited as indicated by lower ALP staining and activity. These results suggested that resveratrol potentiates BMP9-induced osteogenic differentiation of MSCs by activating AMPK-dependent autophagy.

The present study had several limitations. First, we did not explore the mechanism of resveratrol stimulating AMPK phosphorylation. Some studies have suggested that resveratrol epigenetically modifies osteogenic transcription factors, such as Runx2 and SP7, by deacetylation. In the present study, however, we did not investigate acetylated Runx2 and SP7. Thus, it remains unclear whether resveratrol enhances BMP9-induced osteogenesis by directly deacetylating Runx2 and SP7. Moreover, resveratrol did not increase the expression level of SIRT1 in the present study, which may be explained by the high dose and prolonged period of resveratrol treatment due to the underlying negative feedback system that regulates excessive sirtuin activity. Finally, we did not investigate the effect of resveratrol on BMP9-inhibited osteoclastogenesis. Additional studies will be required to elucidate the detailed mechanism of the synergistic function of resveratrol and BMP9 on osteogenesis.

## 5. Conclusion

In conclusion, the present study demonstrated that resveratrol potentiates BMP9-induced osteogenesis by activating the AMPK/Autophagy pathway.

## Data Availability

The original contributions presented in the present study are included in the article and supplementary material. Further inquiries can be directed to the corresponding author.

## Supplementary Materials

Supplementary Materials Supplementary Materials Additional figures contained in supplementary materials that are Figure S1, Figure S2, Figure S3, and Figure S4 are results described in Results and indispensable to support the conclusion. Figure S1: WB analysis of the endogenous expression of BMP9 in C3H10T1/2 cells after Ad-BMP9 infection and Sirt1 and p-mTOR levels in the presence or absence of Ad-BMP9 and Resveratrol. Figure S2: WB analysis of siRNA effect after transfection of siAMPK*α* and siBeclin1. Figure S3: the ALP analysis and WB analysis to support the result that Com.C is able to suppress the osteogenic effect of resveratrol. Figure S4: the ALP analysis and WB analysis to confirm that siBeclin1 is able to inhibit the osteogenic effect of resveratrol. All supplementary images are additional tests for Figure 5.
