# Supplementary material for: Resveratrol Synergistically Promotes BMP9-Induced Osteogenic Differentiation of Mesenchymal Stem Cells
Source: Stem Cells Int. 2022 Jul 25;2022:8124085. doi: 10.1155/2022/8124085 (PMC9343184; doi:10.1155/2022/8124085)
Supplement: Supplementary Materials — Additional figures contained in supplementary materials that are Figure S1, Figure S2, Figure S3, and Figure S4 are results described in Results and indispensable to support the conclusion. Figure S1: WB analysis of the endogenous expression of BMP9 in C3H10T1/2 cells after Ad-BMP9 infection and Sirt1 and p-mTOR levels in the presence or absence of Ad-BMP9 and Resveratrol. Figure S2: WB analysis of siRNA effect after transfection of siAMPKα and siBeclin1. Figure S3: the ALP analysis and WB analysis to support the result that Com.C is able to suppress the osteogenic effect of resveratrol. Figure S4: the ALP analysis and WB analysis to confirm that siBeclin1 is able to inhibit the osteogenic effect of resveratrol. All supplementary images are additional tests for Figure 5. [file 8124085.f1.doc]

**Supplementary Materials**


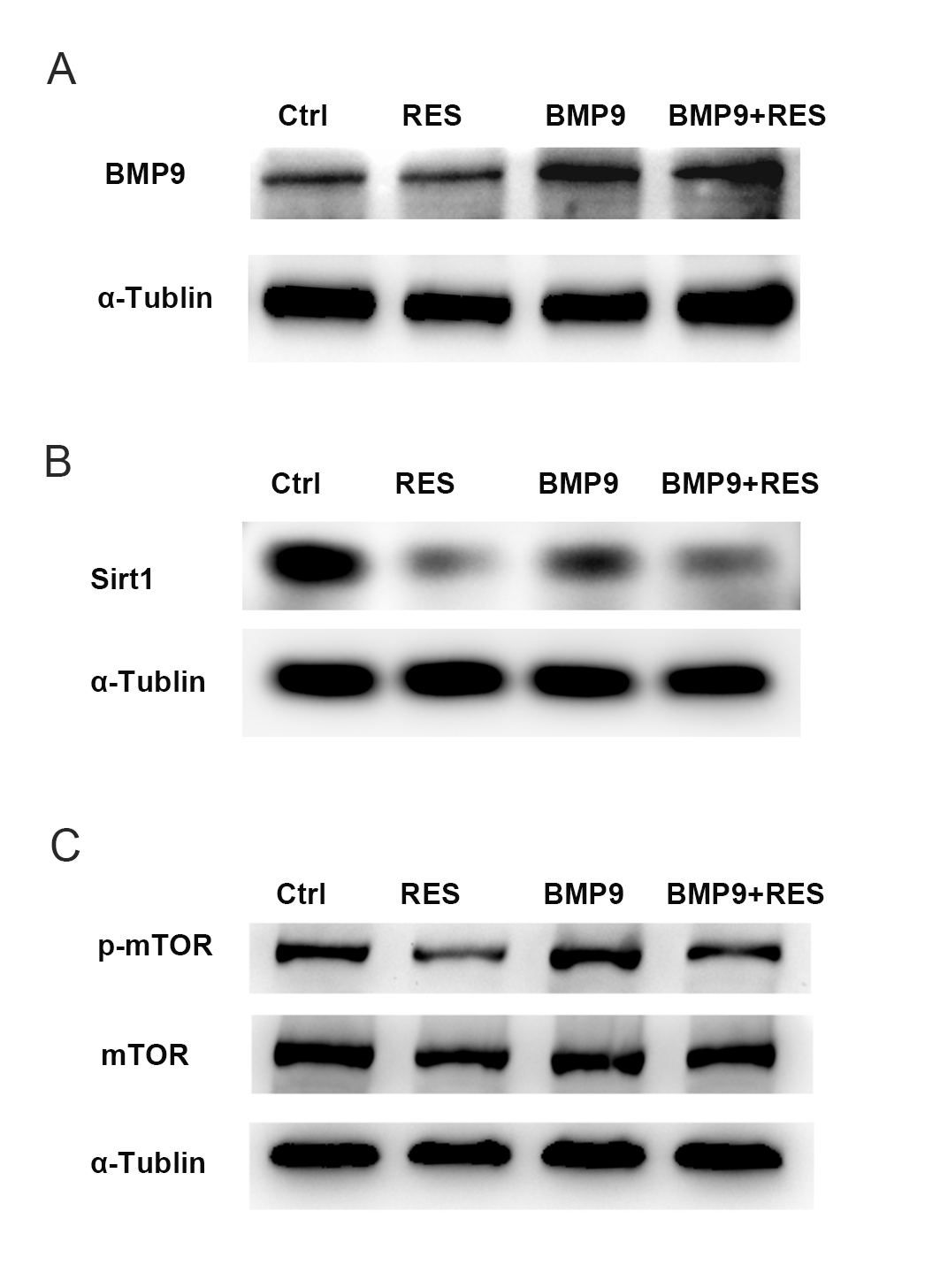


Figure S1. **(A)** WB analysis of the endogenous expression of BMP9 in C3H10T1/2 cells after Ad-BMP9 infection. **(B)** WB analysis showed that resveratrol did not increase the SIRT1 levels in the control and BMP9 groups. **(C)** WB analysis showed that resveratrol treatment suppressed *p*-mTOR. **p*＜0.05, ** *p*＜0.01 and *** *p*＜0.001 compared to the control group. #*p*＜0.05, ## *p*＜0.01, and ### *p*＜0.001 compared with the BMP9 group.


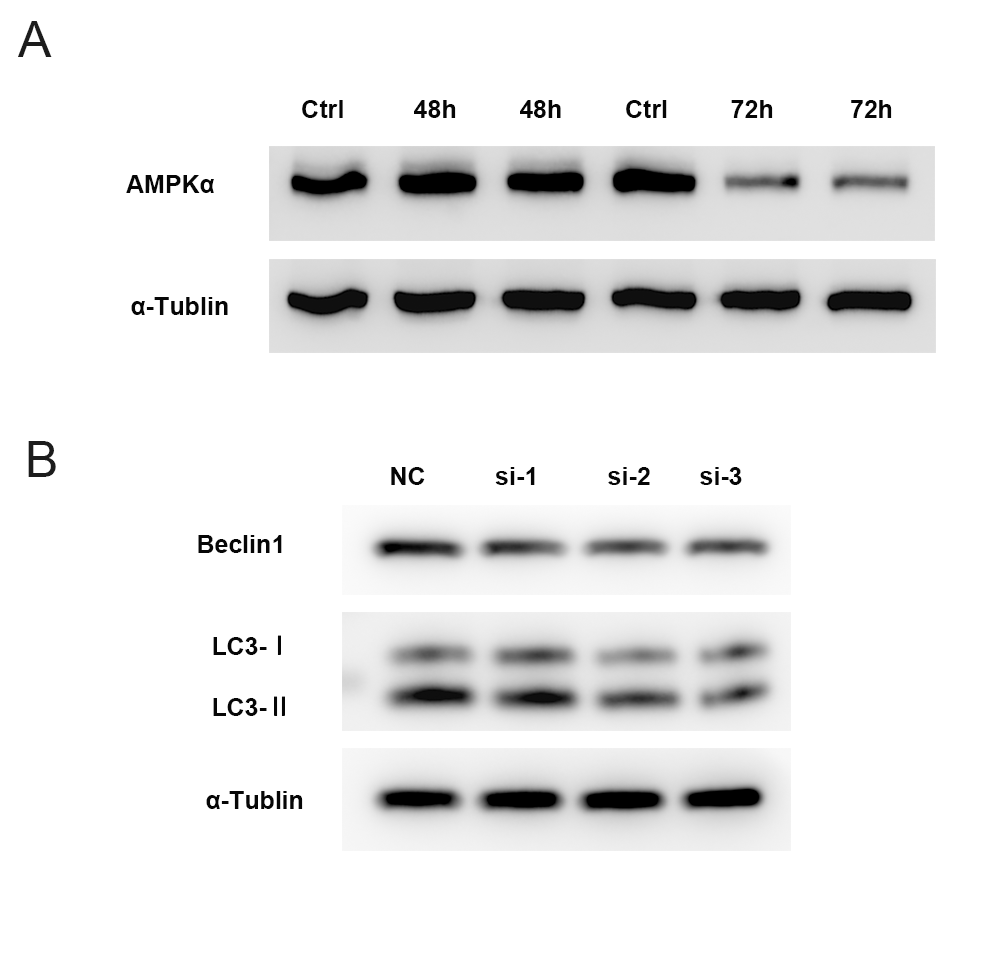


Figure S2. **(A)** Changes in AMPK protein levels 48 h and 72 h after transfection. **(B)** siBeclin1 diminished autophagy in C3H10T1/2 cells.


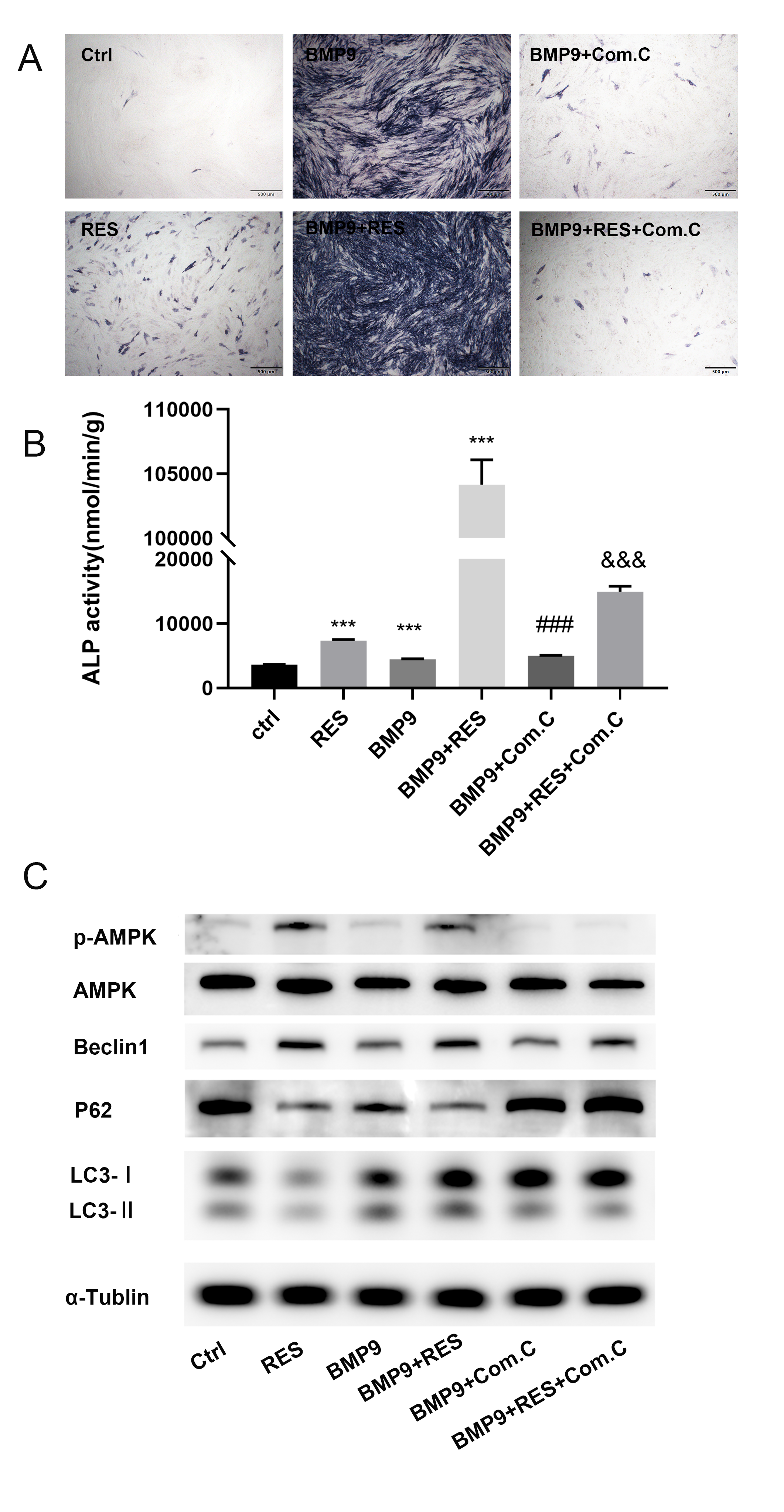


Figure S3. **(A, B)** Compound C, an AMPK inhibitor, abolishes resveratrol-induced osteogenesis. After treatment with the combination of compound C and Ad-BMP9 or Ad-BMP9+resveratrol for 7 d, ALP staining **(A)** and ALP activity assays **(B)** were utilized to evaluate osteogenesis. **(C)** Compound C inhibited the resveratrol-induced phosphorylation of AMPK. After treatment with compound C and Ad-BMP9 in the presence or absence of resveratrol for 72 h, protein levels were evaluated by WB analysis. **p*＜0.05, ** *p*＜0.01 and *** *p*＜0.001 compared to the control group. #*p*＜0.05, ## *p*＜0.01, and ### *p*＜0.001 compared with the BMP9 group. &*p*＜0.05, && *p*＜0.01, and &&& *p*＜0.001 compared to the BMP9+resveratrol group.


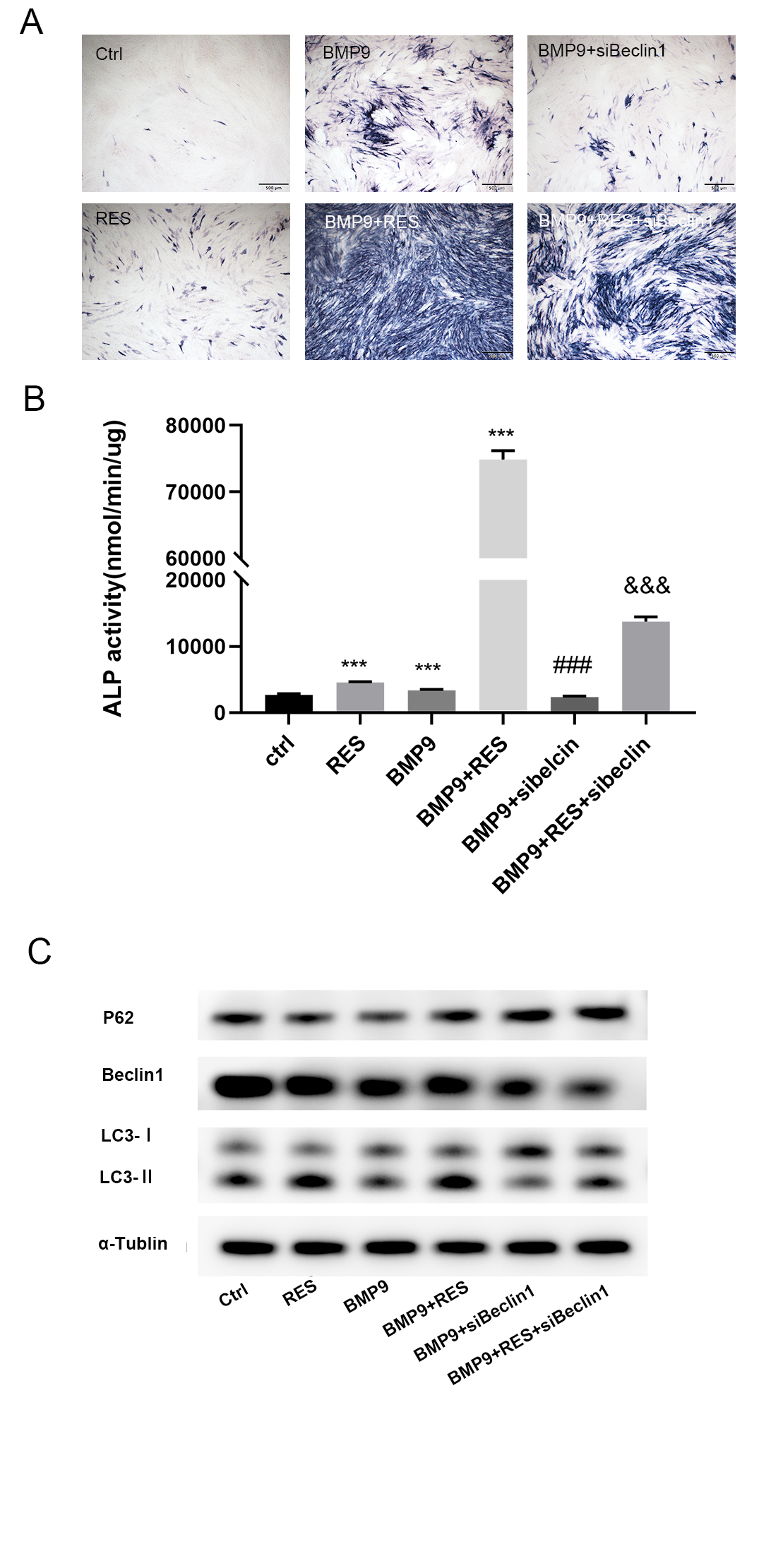


Figure S4. **(A, B)** Downregulation of Beclin1 inhibits the osteogenic induction ability of resveratrol with or without Ad-BMP9. C3H10T1/2 cells were transfected with siBeclin1 24 h prior to treatment with Ad-BMP9 in the presence or absence of resveratrol. After 7 d of osteogenic differentiation, ALP staining **(A)** and ALP activity assays **(B)** were performed to evaluate osteogenesis. **(C)** Transfection of siBeclin1 inhibited resveratrol-induced autophagy. After treatment of C3H10T1/2 cells with siBeclin1 and Ad-BMP9 or Ad-BMP9+resveratrol for 48 h, cell lysates were collected to measure the protein levels of LC3, Beclin1 and p62 by WB analysis. **p*＜0.05, ** *p*＜0.01 and *** *p*＜0.001 compared to the control group. #*p*＜0.05, ## *p*＜0.01, and ### *p*＜0.001 compared with the BMP9 group. &*p*＜0.05, && *p*＜0.01, and &&& *p*＜0.001 compared to the BMP9+reseveratrol group.
